# TCR repertoire profiling revealed antigen-driven CD8+ T cell clonal groups shared in synovial fluid of patients with spondyloarthritis

**DOI:** 10.3389/fimmu.2022.973243

**Published:** 2022-10-17

**Authors:** Ekaterina A. Komech, Anastasia D. Koltakova, Anna A. Barinova, Anastasia A. Minervina, Maria A. Salnikova, Evgeniya I. Shmidt, Tatiana V. Korotaeva, Elena Y. Loginova, Shandor F. Erdes, Ekaterina A. Bogdanova, Mikhail Shugay, Sergey Lukyanov, Yury B. Lebedev, Ivan V. Zvyagin

**Affiliations:** ^1^ Department of Genomics of Adaptive Immunity, Shemyakin-Ovchinnikov Institute of Bioorganic Chemistry, Moscow, Russia; ^2^ Department of Molecular Technologies, Institute of Translational Medicine, Pirogov Russian National Research Medical University, Moscow, Russia; ^3^ Department of Systemic Sclerosis, Nasonova Research Institute of Rheumatology, Moscow, Russia; ^4^ Department of Immunology, St. Jude Children’s Research Hospital, Memphis, TN, United States; ^5^ Department of Rheumatology, Pirogov City Clinical Hospital #1, Moscow, Russia; ^6^ Department of Spondyloarthritis, Nasonova Research Institute of Rheumatology, Moscow, Russia

**Keywords:** TCR repertoire, spondyloarthritis, synovial fluid, ankylosing spondylitis, psoriatic arthritis

## Abstract

Spondyloarthritis (SpA) comprises a number of inflammatory rheumatic diseases with overlapping clinical manifestations. Strong association with several HLA-I alleles and T cell infiltration into an inflamed joint suggest involvement of T cells in SpA pathogenesis. In this study, we performed high-throughput T cell repertoire profiling of synovial fluid (SF) and peripheral blood (PB) samples collected from a large cohort of SpA patients. We showed that synovial fluid is enriched with expanded T cell clones that are shared between patients with similar HLA genotypes and persist during recurrent synovitis. Using an algorithm for identification of TCRs involved in immune response we discovered several antigen-driven CD8+ clonal groups associated with risk HLA-B*27 or HLA-B*38 alleles. We further show that these clonal groups were enriched in SF and had higher frequency in PB of SpA patients vs healthy donors, implying their relevance to SpA pathogenesis. Several of the groups were shared among patients with different SpAs that suggests a common immunopathological mechanism of the diseases. In summary, our results provide evidence for the role of specific CD8+ T cell clones in pathogenesis of SpA.

## 1 Introduction

Spondyloarthritis (SpA) comprises several inflammatory rheumatic diseases with overlapping clinical manifestations, including ankylosing spondylitis (AS), psoriatic arthritis (PsA), reactive arthritis, enteropathic SpA, and undifferentiated SpA. The prevalence of SpA ranges from 0.01 to 2.5% in different human populations (1). To date no SpA specific therapy exists, and treatment involves a variety of anti-inflammatory and immunosuppressive drugs. Still the therapy is often not effective or needs life-long administration as more than 50% of patients relapse upon drug withdrawal (2–4). Genetic factors contribute significantly to the risk of different SpAs, among which association with several HLA class I alleles is the strongest. Two of the most frequent risk alleles are HLA-B*27 and HLA-B*38: their prevalence varies in different populations and accounts for 89% and 1.3% in AS patients, and 20% and 3.1-7.2% in PsA patients, respectively (5–10). Genetic associations of SpA also include other components of the antigen-presenting system (e.g. ERAP1/2) and immune response regulation (e.g. IL23R, IL6R, STAT3) (5). Considering accumulation of T lymphocytes in inflamed joints (11), this suggests the involvement of T cells in pathogenesis of SpA.

T cell receptor (TCR) repertoire profiling provides valuable data to study immune response and identify condition-associated T cell clones (12). Recently we and others discovered a group of T cells clones with highly similar TCRbeta that was overrepresented in peripheral blood (PB) of HLA-B*27+ AS patients (13–15). This TCRbeta motif was previously reported in synovial fluid (SF) of HLA-B*27+ patients with reactive arthritis (16), and further detected in SF of AS patients (13). A number of studies also demonstrated the presence of different clonal T cell expansions in inflamed joints of PsA patients (17–19). Investigation of T cell repertoire of an inflamed site provides insight into the role of T cells in local inflammation and allows identification of the disease-associated clonal expansions that may serve as both diagnostic markers and therapy targets. However, previous studies in this area were limited either by the cohort size or by repertoire sequencing depth.

Here we performed high-throughput TCRbeta repertoire profiling of synovial fluid collected once or twice from a total of 28 patients with AS or PsA and identified on average 28,000 distinct clonotypes per sample. We analyzed general characteristics of SF clonal repertoire, its diversity, sharing and convergence, and investigated the existence of specific T cell clonal expansions restricted to SpA-associated HLA-I alleles.

## 2. Materials and methods

### 2.1. Patients and samples

The study was conducted in accordance with the Declaration of Helsinki and approved by the local ethics committee of Pirogov Russian National Research Medical University. All patients gave their written informed consent for participation. We collected 28 SF and 51 PB samples from patients with PsA (n=17) or AS (n=35). Patients were classified with ankylosing spondylitis or psoriatic arthritis according to modified New-York criteria or CASPAR, respectively (20, 21). For 4 patients additional SF samples were collected at time of relapse: two patients relapsed upon therapy discontinuation, while for the other two patients the therapy was ineffective. Details on the cohort are provided in **Table 1** and **Supplementary Table 1**.

**Table 1:** Patient cohort characteristics.

	PsA n = 17	AS n = 35	Combined n = 52
Male sex, n (%)	12 (71%)	29 (83%)	41 (79%)
Age, years, mean ± sd	37.43 ± 8.77	33.85 ± 7.6	34.90 ± 8.03
Disease duration, years, mean ± sd	8.4 ± 6.11	12.9 ± 8.15	11.3 ± 7.7
HLA-B*27+ donors, n (%) *	7 (41%)	30 (86%)	37 (71%)
HLA-B*38+ donors, n (%) *	7 (41%)	8 (23%)	15 (29%)
HLA-B*27+/B38+ donors, n (%)	0	5 (14%)	5 (10%)

*Including HLA-B*27+/B*38+ donors.

PsA, psoriatic arthritis; AS, ankylosing spondylitis; HLA, human leukocyte antigen; sd, standard deviation.

### 2.2. Isolation of T cell subsets

To isolate mononuclear cells (MNCs) from PB and SF samples we diluted the samples 4 times with 1x PBS and proceeded with Ficoll density gradient centrifugation (PanEco). CD4+ and CD8+ T cells were isolated immediately from MNCs with anti-CD4 or anti-CD8 positive-selection magnetic beads (Dynabeads, Thermo Fisher Scientific). The PD-1+ T cells were sorted from fresh SF MNCs, and CD137+ T cells and CD103+CD69± T cells (tissue-resident memory) were sorted from cryopreserved SF MNCs using the following antibodies: CD3-PE/Cy5 (Beckman Coulter Cat# IM2635U, RRID : AB_10645166), CD8-PE/Cy5 (Beckman Coulter Cat# IM2638U, RRID : AB_131157), CD3-eFluor 450 (Thermo Fisher Scientific Cat# 48-0038-80, RRID : AB_1518801), PD-1-Brilliant Violet 421 (BioLegend Cat# 329920, RRID : AB_10960742), CD137-PE (Miltenyi Biotec Cat# 130-110-900, RRID: AB_2654985), CD103-FITC (Thermo Fisher Scientific Cat# 11-1031-81, RRID : AB_465175), CD69-PE/Cy5 (BioLegend Cat# 310907, RRID : AB_314842). The Helix NIR stain (Thermo Fisher Scientific) was used to exclude dead cells. Cell sorting was performed on FACSAria III (BD) directly into the RLT lysis buffer (Qiagen). Gating strategies are provided in **Supplementary Figure 1**.

### 2.3. TCRbeta library preparation, HLA-typing, and sequencing

Libraries of TCRalpha and TCRbeta chains were prepared with the protocol that implements double barcoding of samples and labeling of cDNA with unique molecular identifiers, allowing for potential cross-sample contamination removal, error-correction, and data normalization [described in (22)]. In brief, total RNA from MNCs was isolated using TRIzol reagent (Thermo Fisher Scientific) or RNeasy Mini kit (Qiagen). Total RNA was used for cDNA synthesis using 5’RACE template switch technology to introduce universal primer binding site, unique molecular identifiers (UMI) and first sample barcode at the 5’ end of RNA molecules. Primers complementary to TCRalpha and TCRbeta constant segments were used for initiation of cDNA synthesis. cDNA was amplified in two subsequent PCR steps. During the second PCR step, second sample barcode and adapters were introduced to the libraries.

The libraries were sequenced on NextSeq, HiSeq2000/2500 or MiSeq (Illumina) with 2x100bp or 2x150bp sequencing length.

HLA-genotyping was performed using an in-house NGS-based protocol. In brief, the total RNA was used to produce the cDNA of the most variable parts of the HLA-A/-B/-C and HLA-DQB/-DRB1/-DRB3/-DRB4/-DRB5 loci. The sequencing was performed on Illumina MiSeq in paired-end mode with 250bp read length. Then we aligned the sequencing data to the IMGT/HLA database (https://www.ebi.ac.uk/ipd/imgt/hla/) and annotated the alleles. The methodology allows typically for resolution to the 3rd field (http://hla.alleles.org/nomenclature/naming.html), thus distinguishing differences on a protein level.

### 2.4. Data analysis and statistics

#### 2.4.1. Raw data preprocessing

Raw sequencing reads were demultiplexed and clustered by UMI using MIGEC software (RRID : SCR_016337, version 1.2.9) with overseq threshold of 2 reads per UMI to exclude erroneous sequences (23). To extract the TCR beta clonotypes from sequencing data the MiXCR software (RRID : SCR_018725, version 3.0.9) was used with settings for 5’RACE paired-end libraries (24). We also used previously published repertoire data of AS patients and healthy donors (13, 25–27). TCR repertoire analysis was performed using tcR R-package and custom R-scripts (28, 29).

#### 2.4.2. Repertoire diversity and clonal sharing

To assess the diversity metrics and clonal sharing all repertoires were normalized by sampling of 34,000 UMIs. Oligoclonality was measured by the Gini index that equals 0 in the even distribution of clonal frequencies and 1 in the monoclonal sample. Clonal sharing was calculated as the median of intersections (i.e., normalized number of TCRbeta clonotypes with identical TRBV, TRBJ and CDR3 amino acid sequence) per donor. Only paired PB and SF samples were included in the analysis (n(Total)=16, n(CD4+)=12, n(CD8+)=12). A two-sided Wilcoxon’s signed-rank test was used to compare the groups. To compare clonal sharing depending on the matches of HLA alleles we considered alleles of HLA-A, -B, -C, -DQB, -DRB1, -DRB3, -DRB4, and -DRB5 loci on level of protein-coding sequence (i.e. at 2nd field resolution). The matches of HLA-DRB3, -DRB4 and -DRB5 loci were counted independently.

#### 2.4.3. Identification of antigen-driven clonal expansions

The ALICE algorithm (Antigen-specific Lymphocyte Identification by Clustering of Expanded sequences) was used to detect clonotypes with a significantly higher number of similar TCRs than expected in the absence of clonal expansion (as predicted by the model of V(D)J-recombination) (30, 31). For estimation of generation probability we run 25 iterations with 5*10^6 recombinations per iteration. Thus, the total number of simulated TCRbeta or TCRalpha sequences (both in-frame and out-of-frame) was 1.25*10^8. Clonotypes represented by 1 UMI were excluded from the repertoires prior to analysis. A significance threshold of Benjamini-Hochberg adjusted p<0.001 was used. Then the significant clonotypes of all donors were clustered in a similarity network based on identity of V- and J-segments and CDR3 length allowing for 1 mismatch in CDR3 amino acid sequence.

#### 2.4.4. Association of TCRbeta motifs with HLA alleles

From the clusters of significant clonotypes resulting from ALICE we selected 29 (**Supplementary Table 3**) based on occurrence of a cluster in at least 3 donors. Occurence of a cluster was calculated as a cumulative occurrence of clonotypes comprising the cluster. To identify clusters associated with HLA-B*27 or HLA-B*38 we compared occurrence of the clusters between CD8+ SF samples of HLA-B*27+ and HLA-B*38+ patients (excluding 1 HLA-B*27+/B*38+ patient) by Fisher’s exact test with Benjamini-Hochberg adjustment.

#### 2.4.5. Annotation with VDJdb

To annotate clonotypes with known antigen-specificity we used the VDJdb database (build 2021-02-02) (32). To exclude ambiguous sequences from the VDJdb it was filtered as follows: among clonotypes with identical CDR3 amino acid sequence annotated as specific to different epitopes we retained those with maximal VDJdb score, maximal number of publications and specificity to a single epitope within a species. All clonotypes with the score = 0 were excluded.

The clonotype was annotated as epitope-specific if its CDR3 amino acid sequence and V-segment, as well as HLA allele (at 2 digit resolution) carried by the donor were identical to those of the corresponding VDJdb record.

The proportion of the T cells occupied by clones specific to EBV epitopes in paired PB and SF samples was compared by two-sided Wilcoxon signed-rank test.

## 3. Results

### 3.1. Synovial fluid of SpA patients contains expanded T cell clonotypes shared among donors with similar HLA genotypes

To study the TCRbeta repertoire in inflamed joints of patients with SpA we collected PB and SF samples from 10 AS and 17 PsA patients. For PB as well as SF samples we isolated at least 8x10^6 total MNCs, of which 3x10^6 MNCs were used to obtain total TCR repertoires, and another 3x10^6 MNCs were used to isolate CD4+ and CD8+ T cell subsets and further reconstruct their TCR repertoires. We also included repertoires of 25 AS patients sequenced for previous work to the analysis (13), thus, collectively analyzing SF and PB samples from 28 and 51 donors, respectively (**Table 1**, **Supplementary Table 1**). All TCRbeta repertoires were reconstructed using the protocol that implements double barcoding of samples and labeling of cDNA with unique molecular identifiers. The median number of unique TCRbeta cDNA molecules identified per sample was 267,258 for PB (IQR 144,492 - 483,323) and 207,539 for SF (IQR 83,554 - 358,229), reflecting comparable repertoire analysis depth of on average ~250,000 T cells (**Supplementary Table 2**) (33).

According to flow cytometry on average ~80% of SF T cells expressed PD-1 and 3-5% T cells expressed CD137 in contrast to less than 10% and 0.5% in PB, respectively (**Supplementary Figure 2**). Thus, T cells accumulated in SF show hallmarks of recent activation by cognate antigens.

**Figure 1:**
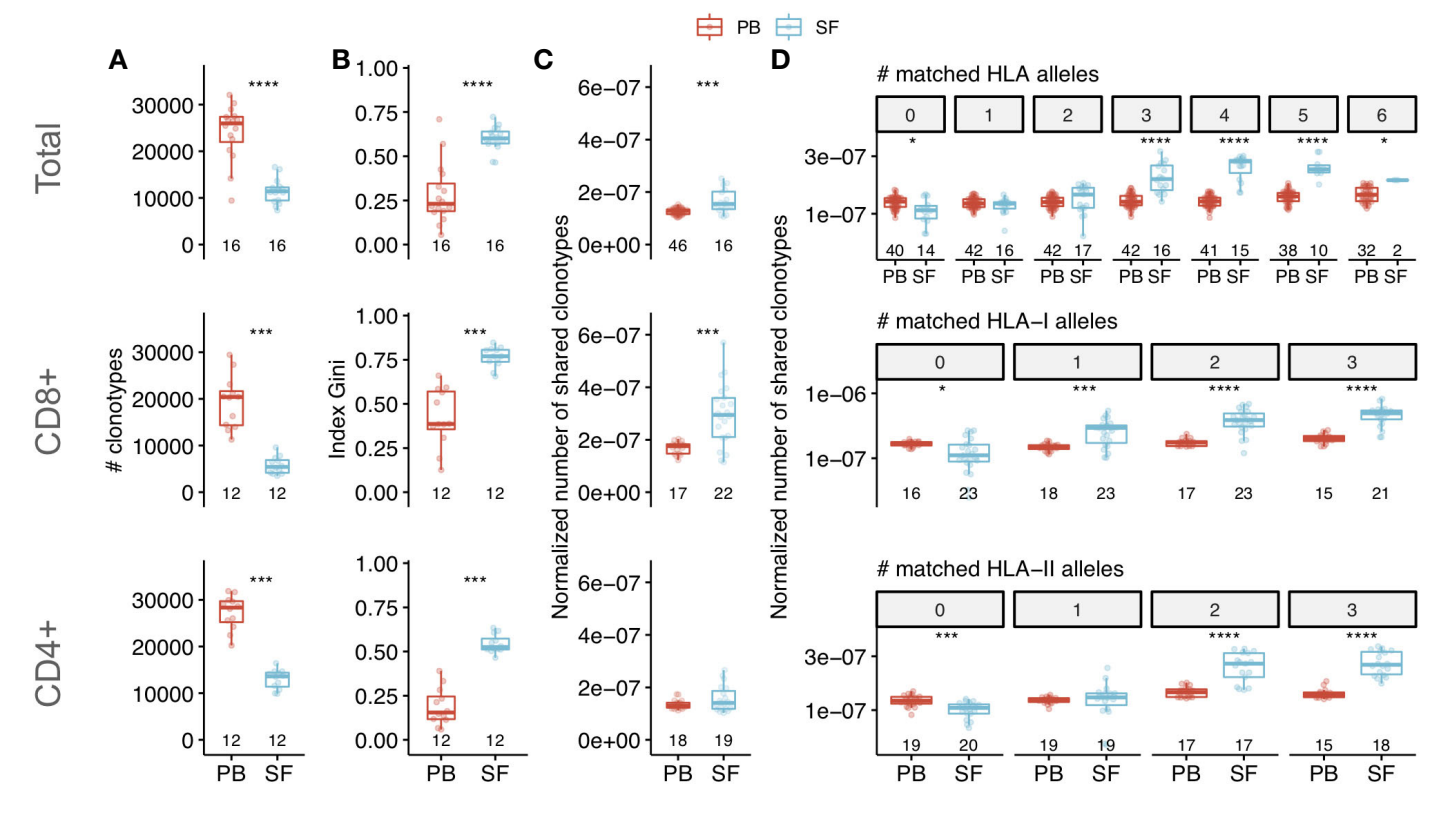
Characteristics of clonal T cell repertoire of synovial fluid from patients with SpA. Comparison of clonal diversity (species richness) **(A)** and oligoclonality **(B)** between PB and SF repertoires of SpA patients (by Wilcoxon signed-rank test). Oligoclonality is measured by Gini index that equals 0 in even distribution of frequencies and 1 in monoclonal sample. **(C, D)** Normalized number of shared clonotypes (i.e., having identical CDR3 amino acid sequence and TRBV-segment) per donor in total **(C)** or depending on the number of matched HLA alleles **(D)** between donors (Wilcoxon rank sum test). Each dot represents an individual sample. For patients with relapses only repertoires of time point 1 were included in the analysis. Values below the box plots denote the number of donors. The middle line of a box corresponds to the median, the lower and upper hinges correspond to the 25th and 75th percentiles, respectively. The upper and lower whiskers extends from the hinges to the largest and smallest values no further than 1.5 * IQR from the corresponding hinge. Top, middle and bottom panels correspond to the repertoires of total, CD8+ and CD4+ T cells respectively. To assess diversity and clonal sharing repertoires were *in silico* equalized to the same sequencing depth. PB - peripheral blood, SF - synovial fluid, HLA - human leukocyte antigen. *p < 0.05, ***p < 0.001, ****p < 0.0001.

To characterize the structure and uniqueness of SF repertoire we compared the clonal diversity, clonal size distribution and repertoire overlap between SF and PB samples. As the diversity metrics and clonal sharing are extremely affected by sample size (34), we equalized the repertoire analysis depth by sampling the 34,000 unique TCRbeta cDNAs from each dataset. Both CD8+ and CD4+ SF repertoires had significantly lower clonal diversity and larger clonal expansions compared with those of PB (**Figures 1A, B**). On average only ~6% of PB clonotypes, defined by CDR3 nucleotide sequence and TRBV and TRBJ segments, were present in matched SF sample independent of CD4 or CD8 lineage.

The total and CD8+ SF repertoires had higher clonal sharing (i.e., number of TCRbeta clonotypes with identical CDR3 amino acid sequence and TRBV and TRBJ segments) between individuals compared with those of PB (**Figure 1C**). Moreover, clonal sharing depended on the number of HLA alleles matched between donors: SF repertoires shared more clonotypes compared with PB only if donors shared at least one HLA-I allele (for CD8+ T cells) or two HLA-II alleles (for CD4+ T cells) (**Figure 1D**). Collectively, these data demonstrate that SF has a distinct structure of clonal repertoire and contains a specific set of expanded T cell clones. These expansions were shared between unrelated patients with shared HLA alleles, thus suggesting that they represent the convergent immune response to the same or similar antigens.

### 3.2. Identification of clonotypes involved in immune response in synovial fluid

To investigate whether these shared T cell clones accumulated in SF antigen-specifically and to identify putative disease-associated T cell clonal groups we employed the ALICE algorithm (30, 31). The algorithm detects co-presence of highly similar clonotypes typically arising during an immune response to the same antigen in contrast to those resulting from VDJ-recombination by chance.

**Figure 2:**
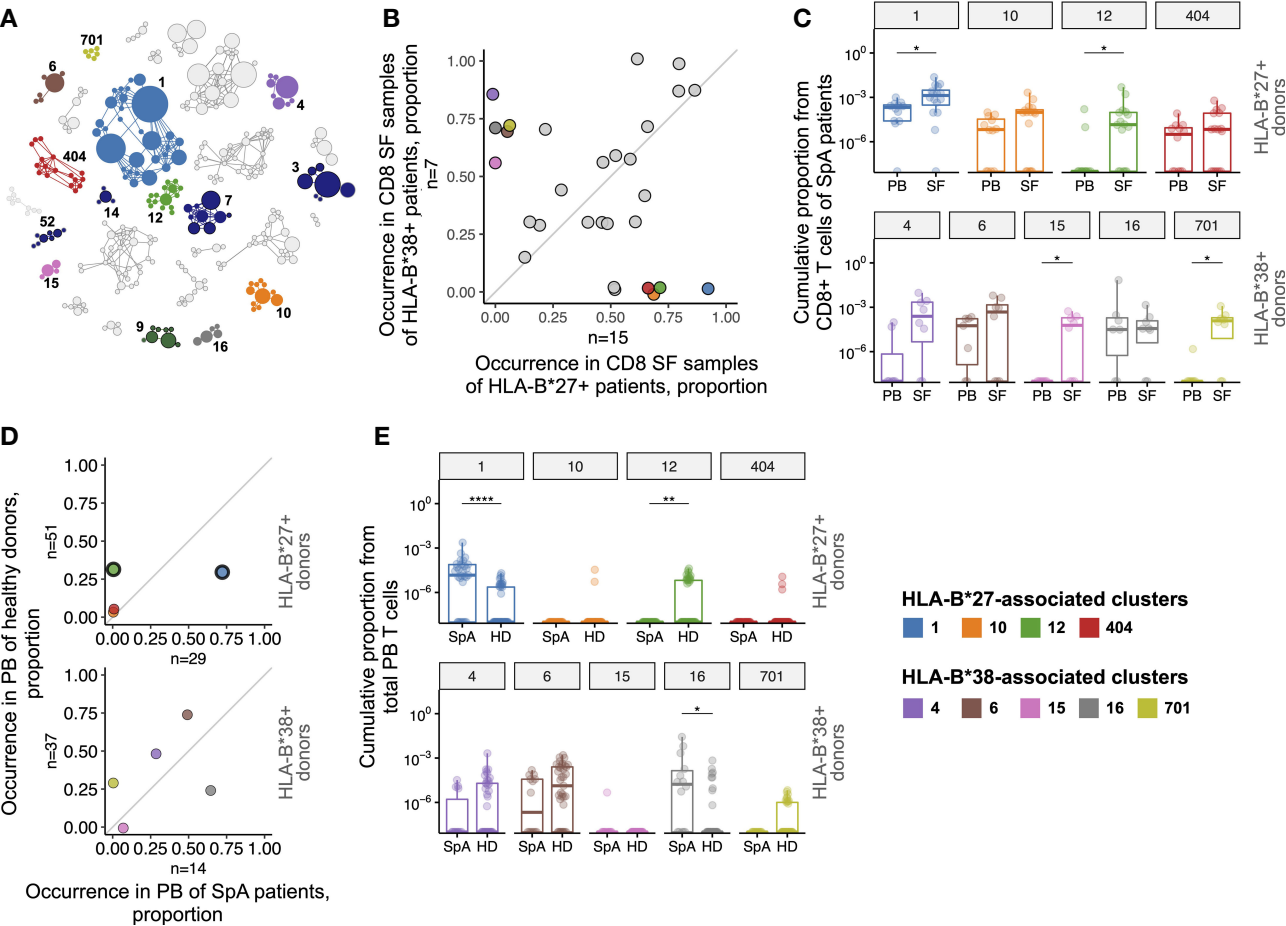
T cell clonotypes involved in immune response in synovial fluid of SpA patients. **(A)**, Similarity network of SF CD8+ clonotypes identified by the ALICE. Only 29 clusters shared by ≥3 patients are shown. Each node represents a unique amino acid clonotype. Edges connect clonotypes differing in one CDR3 amino acid residue. Node’s size corresponds to the number of donors sharing this clonotype. Color highlights clusters associated with HLA-B*27, HLA-B*38, or found in VDJdb. **(B)**, Occurrence of the clusters in CD8+ SF samples of HLA-B*27+ (n=15) vs HLA-B*38+ (n=7) patients. **(C)**, T cell frequency of HLA-B*27-associated (upper, n(PB)=12, n(SF)=16) or HLA-B*38-associated (bottom, n(PB)=7, n(SF)=8) clusters in CD8+ repertoires of patients. Occurrence **(D)** and T cell frequency **(E)** of HLA-B*27-associated (upper, n(SpA)=29, n(HD)=51) or HLA-B*38-associated (bottom, n(SpA)=14, n(HD)=37) clusters in total PB repertoires of SpA patients and HD. Black circles mark clusters significantly enriched by Fisher’s exact test with Benjamini-Hochberg adjusted p<0.05. On the box plots the middle line corresponds to the median, the lower and upper hinges correspond to the 25th and 75th percentiles, respectively. The upper and lower whiskers extends from the hinges to the largest and smallest values no further than 1.5 * IQR from the corresponding hinge. Each dot represents an individual sample. For patients with relapses only repertoires of time point 1 were included in the analysis. PB - peripheral blood, SF - synovial fluid, SpA - spondyloarthritis, HD - healthy donors. Wilcoxon rank sum test with Benjamini-Hochberg adjustment *p < 0.05, **p < 0.01, ****p < 0.0001.

The ALICE was applied to each of the 28 CD8+ SF repertoires separately yielding on average ~300 significant, i.e., having more homologs than expected in the absence of clonal expansion, amino acid clonotypes per sample (median 192, IQR 27-343). We then combined all such clonotypes (n=4432) from all donors and clustered them into similarity networks based on the identity of V-J-segments and CDR3 length allowing 1 amino acid substitution in CDR3 (30–32, 35, 36). Overall we got 1449 clusters: 1377 clusters were present in a single patient, while the others were shared, and 29 of them were shared by at least 3 patients (**Figure 2A**).

Presence of virus-specific T cell clones were described in different inflammatory backgrounds (37, 38). We used the VDJdb, a database of TCRs with known antigen specificity, to search for the clonotypes related to different viral epitopes among clonotypes of the clusters (32). We found that 5 of the 29 shared clusters comprised clonotypes associated with HLA-A*02-restricted epitopes from EBV (clusters 3, 7, 14 and 52) and Influenza A virus (cluster 9). Accordingly, these clusters were detected exclusively in HLA-A*02+ individuals. Notably, the EBV-associated clonotypes were enriched in SF compared with PB samples (**Supplementary Figure 3**), implying accumulation of EBV-specific T cell clones in SF.

We further searched for clusters associated with two SpA risk HLA-I alleles, HLA-B*27:05 and HLA-B*38:01 that were present almost mutually exclusive in our cohort (n=16 and n=8, respectively, including one HLA-B*27+/B*38+ donor; HLA-B*38 was present as a HLA-B*38:01/C*12:03 haplotype). Nine clusters were significantly associated with the presence of these alleles: clusters 1, 10, 12, 404 were overrepresented in CD8+ SF repertoires of HLA-B*27+ patients, and clusters 4, 6, 15, 16, 701 were overrepresented in repertoires of HLA-B*38+ patients (**Figure 2B** and **Supplementary Table 3**).

Clonotypes from cluster 1 matched or were similar to the previously identified AS-related TCRbeta motif (13–15) and were detected in 15 out of 16 CD8+ SF samples of HLA-B*27+ patients irrespective of diagnosis, including HLA-B*27+/B*38+ donor (**Supplementary Table 3**). The most shared clonotype of cluster 4 TRBV10-2_CASSESPGNSNQPQHF_TRBJ1-5 was reported to be HLA-C*12:03-restricted and accordingly was present in CD8+ SF samples of 6 out of 8 HLA-B*38:01+/С*12:03+ patients and 1 out of 3 B*38:01-/C*12:03+ patients (39).

To address the relevance of the identified clusters to the joint inflammation we analyzed their enrichment in SF, as well as the prevalence in PB repertoires of SpA patients in comparison with a large cohort of healthy donors (HD, combined dataset collected from several previously published studies) (13, 25–27). The T cell frequency (i.e., cumulative proportion of T cells occupied by clonotypes of a cluster) of clusters 1, 12, 15 and 701 was significantly higher in CD8+ SF than in PB repertoires of HLA-B*27+ or HLA-B*38+ patients (**Figure 2C**; **Supplementary Figure 4**), implying that the clonotypes specifically accumulated in an inflamed joint. In total PB samples two clusters had significantly higher T cell frequency (clusters 1 and 16) and cluster 1 was also overrepresented in SpA patients compared with HD (**Figures 2D, E**; **Supplementary Table 4**). On the contrary, cluster 12 was barely present in blood of patients but had significantly higher occurrence and T cell frequency in HD. The T cell frequency of the other clusters was either extremely low (clusters 10, 404, 15 and 701), or did not differ (clusters 4 and 6) between PB of HD and SpA patients.

We next analyzed the presence of the clusters in subsets of activated (PD-1+ as well as CD137+) SF T cells and tissue-resident memory (CD103+CD69±) SF T cells of the donors. The clonotypes of the clusters 1, 12, 4, 6, 15 were detected among activated T cells, and clusters 1 and 6 among tissue-resident memory T cells (**Supplementary Table 5**), further supporting their involvement in local inflammation.

TCR alpha chain can also be involved in or determine antigen recognition (40, 41). We applied the same methodology to identify the overrepresented TCR groups in the TCRalpha repertoires of CD8+ SF T cells. The TCRalpha repertoires were reconstructed from the same samples as TCRbeta for 10 SpA patients (5 HLA-B*27+, 4 HLA-B*38+, 1 HLA-B*27+/B*38+). In sum we identified ten clusters, and only one of them was shared among different patients (**Supplementary Figure 5**). This cluster consisted of clonotypes with TRAV1-2/TRAJ33 segments that typically form invariant TCRalpha of mucosal-associated invariant T cells (MAIT). Several clonotypes from the cluster exactly matched the known CDR3alpha of MAIT cells (http://www.cbs.dtu.dk/services/MAIT_Match) (42). Absence of significant TCRalpha clusters (other than MAIT TCRs) indicates that the paired alpha chains of the clonotypes from the identified TCRbeta groups have diverse CDR3s. One also could not exclude that some of the observed TCRbeta clusters may represent MAIT cells.

### 3.3. The same expanded clonotypes are present in an inflamed joint during recurrent synovitis

To study how SF clonal T cell repertoire varies between relapses of synovitis we collected repeated SF samples from 4 SpA patients (9-23 months apart).

The overall similarity of the repertoires at those time points was estimated with Morisita’s overlap index that accounts for both occurrence and T cell frequency of identical clonotypes between samples. The index is bounded between 0 (completely different repertoires) and 1 (absolutely identical repertoires). SF repertoires of the same patient appeared highly similar for all donors (Morisita’s index median 0.759, IQR 0.639 - 0.827, compared with <1*10^-5 for unrelated samples). Furthermore, out of the 1000 most abundant clonotypes at time point 1 more than 800 (median 838.5, IQR 831.5 - 853.2) were also detected at time point 2, and more than 400 (median 464.5, IQR 425.5 - 521.0) remained among the top 1000 clonotypes, indicating the stability of major clonal expansions of SF.

By employing the ALICE algorithm we found that only ~30% of inferred antigen-driven clonal expansions were novel at time point 2 (median 29.7% IQR 27.2% - 31.9%), while the majority of them were shared between the time points. Of the previously identified HLA-B*27-associated clusters the clonotypes of cluster 1 were observed in repeated SF samples of all 3 HLA-B*27+ donors, and clonotypes of clusters 12 and 404 persisted in one of the three donors.

## 4 Discussion

Here we applied high-throughput TCR repertoire profiling to the synovial fluid of a large cohort of SpA patients to characterize the repertoire structure and to identify T cell clones involved in disease pathogenesis. We demonstrated that both CD4+ and CD8+ SF clonal repertoires have low diversity and contain large clonal expansions distinct from those of blood, confirming and extending previous observations (17–19, 43). The cytometry data showed that the majority of SF T cells exhibit signs of TCR-dependent activation in concordance with results obtained with single-cell RNAseq (43). We also showed that the same clonotypes are present in repeated SF samples collected several months apart, suggesting that they persist in the joint as tissue-resident T cells. Proinflammatory CD8+ T cells with resident memory phenotype were recently discovered in SF of PsA patients (43, 44).

**Figure 3:**
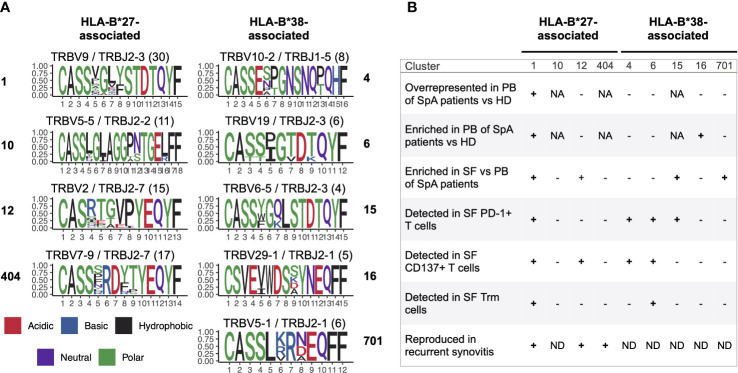
Characteristics of identified TCRbeta clusters. Sequence logo of CDR3 regions **(A)** and details **(B)** of the 9 identified clusters associated with HLA-B*27 or HLA-B*38. Values in parentheses represent the number of distinct clonotypes. PB, peripheral blood; SF, synovial fluid; NA, not applicable; ND, no data.

By comparing repertoires between patients we found that identical clonotypes accumulate in SF of unrelated donors with similar HLA genotypes, suggesting selection of T cell clones specific to the same or similar epitopes that could have self or non-self origin. Considering that the immune response to a single antigen:MHC complex is mediated by T cell clones with highly similar TCRs (30–32, 35, 36), we discovered clusters of similar clonotypes shared between CD8+ SF samples of patients. Among them we identified nine clusters that were associated with SpA risk HLA-I alleles: HLA-B*27 or HLA-B*38. The T cells of these clusters were enriched in synovial fluid compared with blood, expressed markers of TCR-dependent activation and/or tissue residency, and persisted in SF during recurrent synovitis (summarized in **Figure 3**, **Supplementary Table 5**). Taken together these data imply the involvement of the identified groups of TCRbeta clonotypes in joint inflammation in SpA.

One of the HLA-B*27-associated clusters, cluster 1, was detected in SF of ~94% HLA-B*27+ patients and matched TCRbeta motif previously identified in HLA-B*27+ AS and ReA patients (13–16). Here we report the presence of the same motif in SF of HLA-B*27+ patients with PsA, providing evidence for a common immunopathological mechanism of different SpAs. We also identified three new TCRbeta clusters enriched in SF of HLA-B*27+ AS and PsA patients. In HLA-B*27- SpA patients we discovered five potentially pathogenic TCRbeta clusters associated with HLA-B*38.

In agreement with the previous report (37), we found shared clusters assigned to HLA-A*02-restricted epitopes of EBV, and, cumulatively, the EBV-associated clonotypes were enriched in SF (**Supplementary Figure 3**). Note that it is common for virus-specific clones to be associated with T cell infiltration into inflamed sites, e.g. tumor-infiltrating T cells (38). These results draw attention to the potential role of virus-specific T cells in immune response to non-viral antigens.

Repertoire analysis based on TCRbeta sequence similarity and sharing between donors allowed us to identify clonal groups expanded in antigen-dependent manner in comparison with diverse bystander T cell clones (45). However, the association of a TCRbeta clonotype with a cluster could be inaccurate due to unknown paired TCRalpha chain and/or unclear impact of particular amino acid mismatches to the antigen-specificity. Together with heterogeneity of T cell phenotypes within a clone and/or cluster (reported recently for SF of PsA patients) (43) this could affect the frequency and detection of a clonotype in different donors. It can be suggested that the dissimilarity in paired TCRalpha chain or T cell phenotype are related to the higher frequency of cluster 12 in PB of HD compared with SpA patients (**Figures 2C, D**; **Supplementary Figure 4**). Further studies designed to characterize paired TCRalpha repertoire, functional phenotype and antigen-specificity of the T cells belonging to the identified TCRbeta clusters are necessary to clarify their role in SpA pathogenesis.

In summary, we demonstrated that SF of SpA patients contains a distinct set of expanded T cell clones that persist over time. We discovered several TCRbeta motifs associated with risk HLA-I alleles and potentially involved in self-antigen recognition during joint inflammation. Our findings provide evidence for a role of antigen-driven CD8+ T cells in pathogenesis of SpA.

## Data availability statement

The data presented in the study are deposited in the ArrayExpress database, accession number E-MTAB-11498.

## Ethics statement

The studies involving human participants were reviewed and approved by Local Ethical Committee of Pirogov Russian National Research Medical University. The patients/participants provided their written informed consent to participate in this study.

## Author contributions

EK, IZ, and YL developed the concept and designed the study. AK, ES, TK, EL, and SE collected samples and patients’ metadata. EK, AB, EB, and IZ processed the samples, performed sequencing and reconstructed TCR repertoires. AM and MAS performed HLA typing. EK and IZ analyzed and interpreted the data and drafted the manuscript. MS, AM, SL, YL, and IZ critically reviewed the manuscript. IZ supervised the study. All authors read and agreed on the final version of the manuscript.

## Funding

The study was supported by Russian Science Foundation grant №20-75-00041.

## Acknowledgments

We are most grateful to all patients and medical staff for participation in this work.

## Conflict of interest

EL is a member of the Speakers bureau of Janssen. TK is a member of Speakers bureau of: Pfizer, MSD, Novartis, AbbVie, Janssen, Lilly, Celgene, JSC BIOCAD, and Novartis-Sandoz. SE is a member of the Speaker’s bureau of KRKKA, MCB and JSC BIOCAD. SL and IZ provide scientific advice for JSC BIOCAD.

The remaining authors declare that the research was conducted in the absence of any commercial or financial relationships that could be construed as a potential conflict of interest.

## Publisher’s note

All claims expressed in this article are solely those of the authors and do not necessarily represent those of their affiliated organizations, or those of the publisher, the editors and the reviewers. Any product that may be evaluated in this article, or claim that may be made by its manufacturer, is not guaranteed or endorsed by the publisher.
