# Supplementary material for: TCR repertoire profiling revealed antigen-driven CD8+ T cell clonal groups shared in synovial fluid of patients with spondyloarthritis
Source: Front Immunol. 2022 Oct 17;13:973243. doi: 10.3389/fimmu.2022.973243 (PMC9618624; doi:10.3389/fimmu.2022.973243)
Supplement: Supplementary file 1 [file DataSheet_1.pdf]

Supplementary Material

1 Supplementary Figures

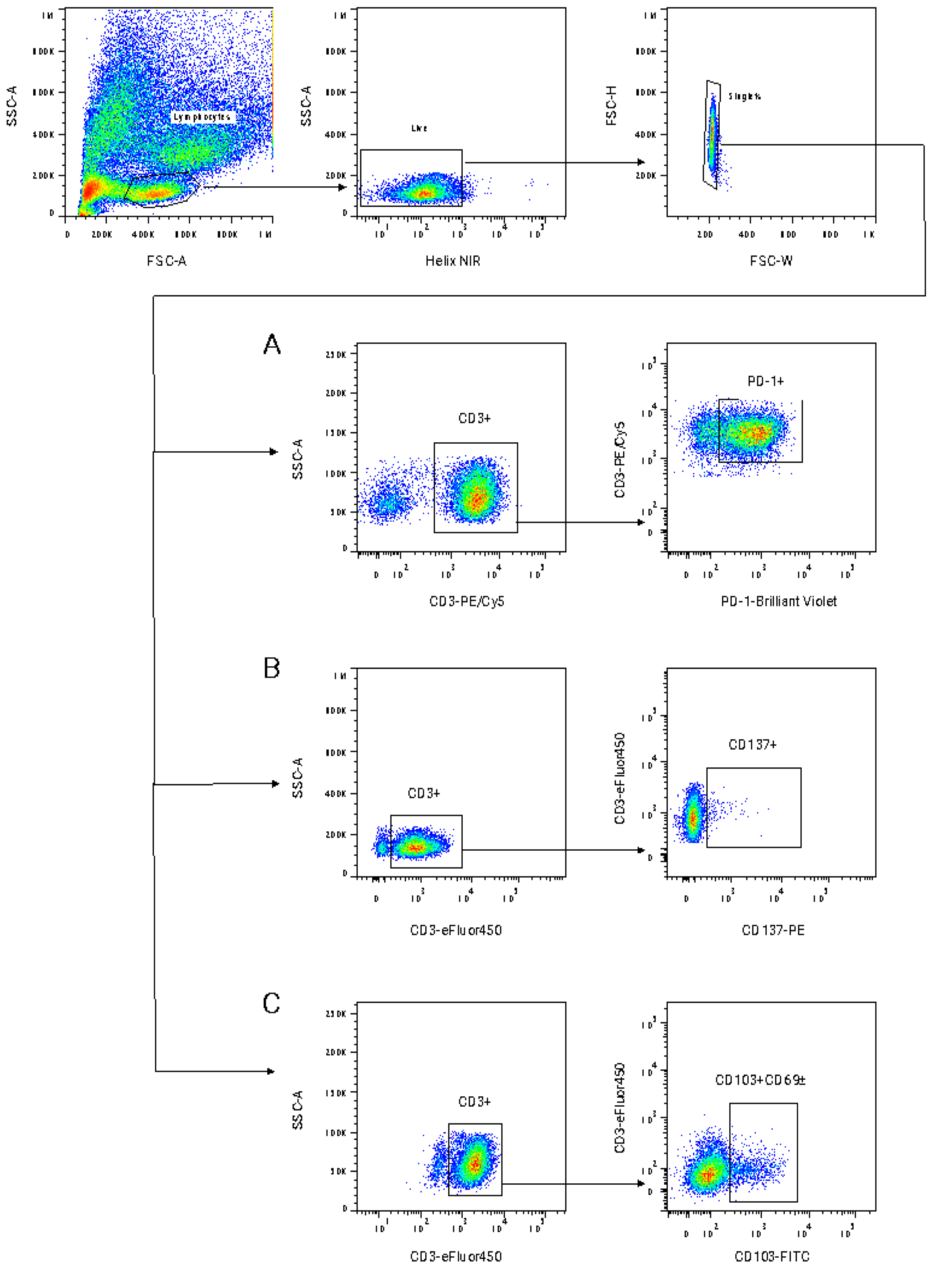

Supplementary Figure 1. Gating strategies for FACS sorting of PD-1+ (A), CD137+ (B) or CD103+CD69± (C) SF T cells.

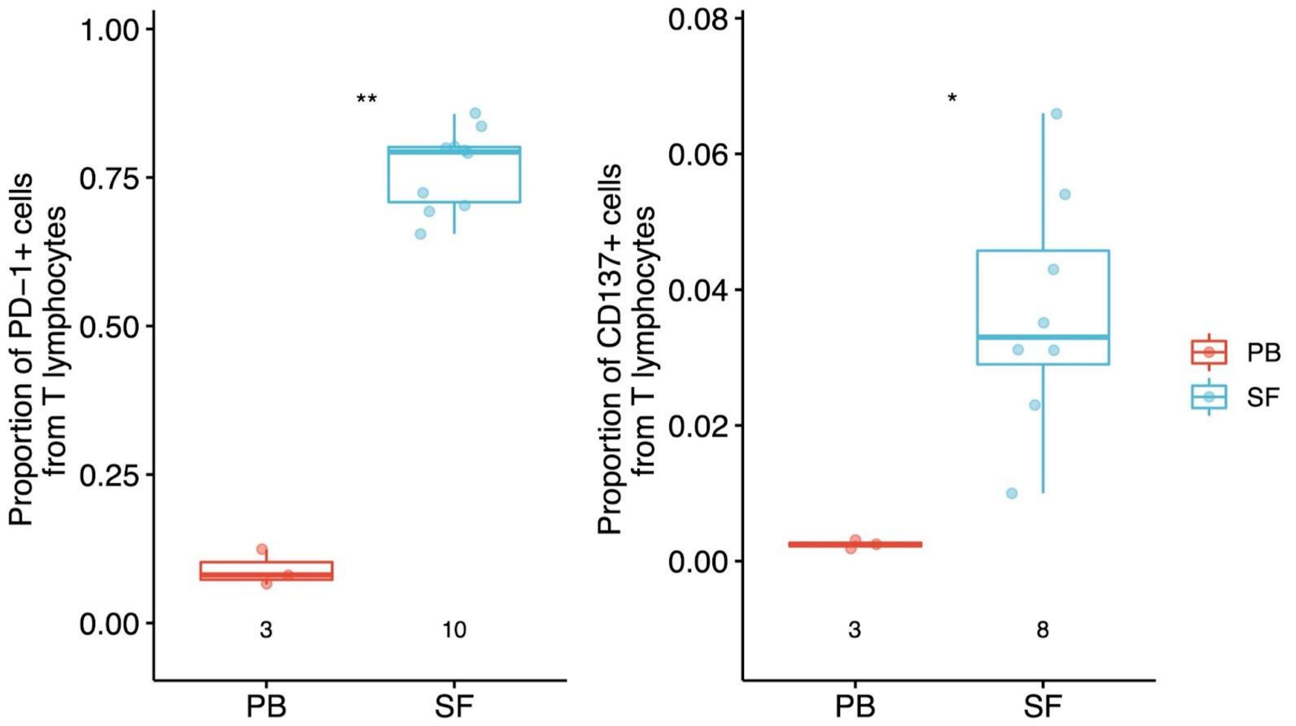

Supplementary Figure 2 . Expression of PD-1 and CD137 on synovial fluid T cells of SpA patients. Proportion of PD-1+ (left) and CD137+ (right) cells from CD3+ cells of peripheral blood (n=3) and synovial fluid (n=10 and n=8 for PD-1 and CD137, respectively) samples. Each dot represents an individual sample. The middle line of the box corresponds to the median, the lower and upper hinges correspond to the 25th and 75th percentiles, respectively. The upper and lower whiskers extends from the hinges to the largest and smallest values no further than 1.5 * IQR from the corresponding hinge. Wilcoxon rank sum test: *p<0.05, **p<0.01.

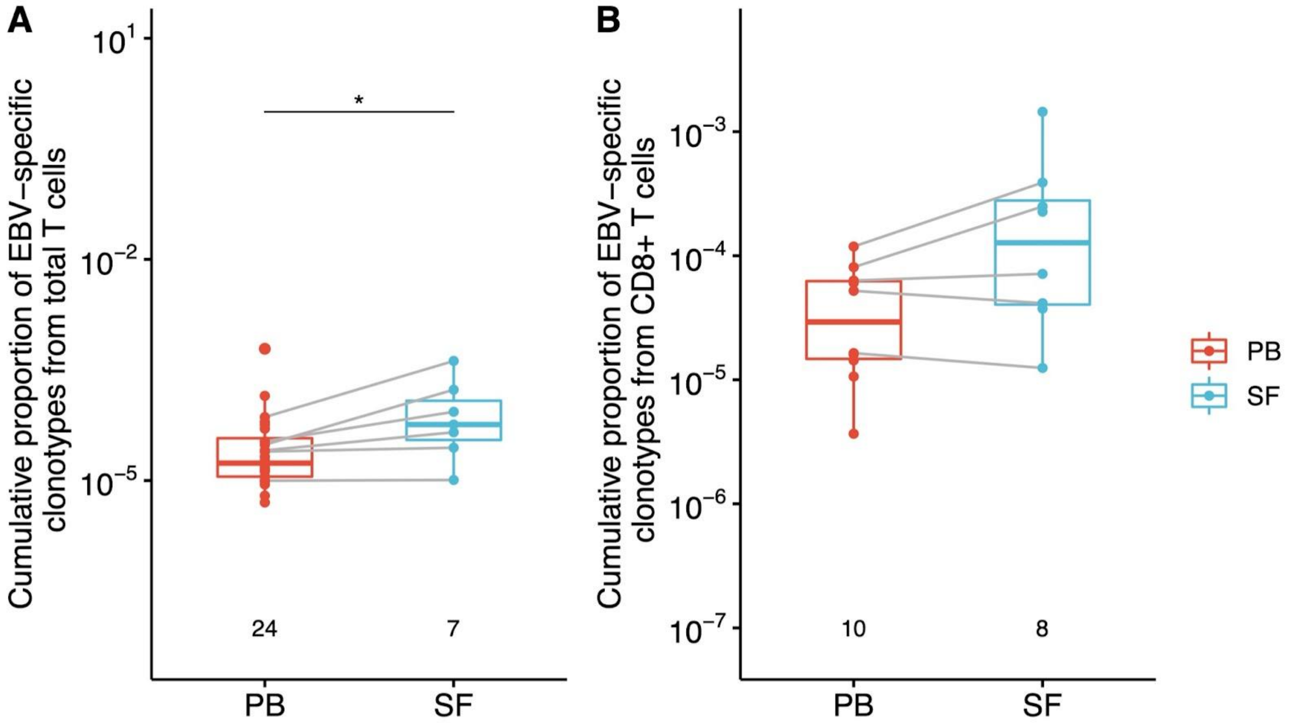

Supplementary Figure 3 . Proportion of EBV-associated T cell clonotypes in SF of SpA patients. Cumulative T cell frequency of clonotypes assigned as specific to EBV epitopes by VDJdb in total (**A**) and CD8+ (**B**) T cell repertoires of SpA patients. Lines connect paired PB and SF samples of the same donor. Each dot represents an individual sample. The middle line of the box corresponds to the median, the lower and upper hinges correspond to the 25th and 75th percentiles, respectively. The upper and lower whiskers extends from the hinges to the largest and smallest values no further than 1.5 * IQR from the corresponding hinge. * Wilcoxon signed-rank test $p<0.05$.

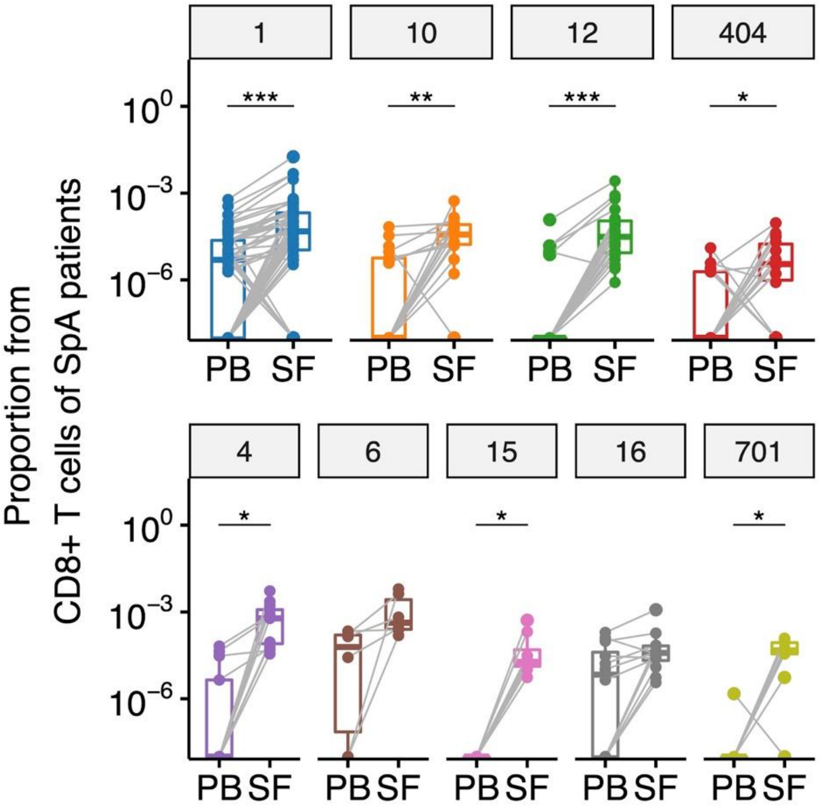

Supplementary Figure 4 . Proportion of clonotypes of identified clusters in paired PB and SF of SpA patients. T cell frequency of clonotypes comprising identified clusters in CD8+ PB and SF repertoires of HLA-B*27+ (upper, n=8) or HLA-B*38+ (bottom, n=5) SpA patients. Points represent amino acid clonotypes, and lines connect the same clonotype within a donor. The middle line of the box corresponds to the median, the lower and upper hinges correspond to the 25th and 75th percentiles, respectively. The upper and lower whiskers extends from the hinges to the largest and smallest values no further than 1.5 * IQR from the corresponding hinge. Wilcoxon signed-rank test with Benjamini-Hochberg adjustment: *p<0.05, **p<0.01, ***p<0.001.

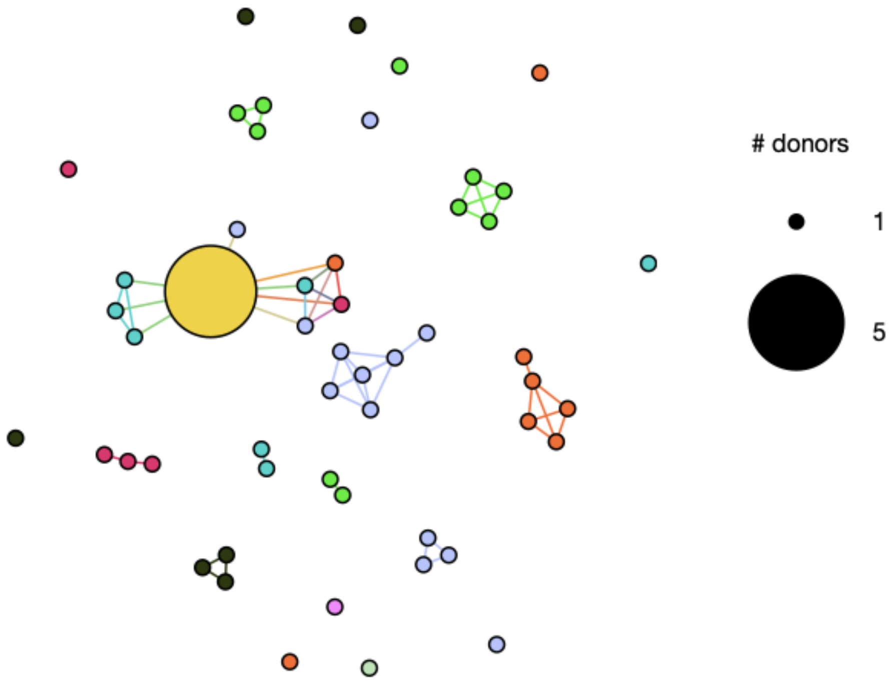

Supplementary Figure 5 . Similarity network of TCRalpha CD8+ SF clonotypes identified by the ALICE. Each node represents a unique amino acid clonotype. Edges connect nodes different in 1 CDR3 amino acid residue. Node's size corresponds to the number of donors sharing this clonotype. Color corresponds to the donor that has this clonotype (except for one shared clonotype, marked yellow).
